# Human Coronavirus NL63, France

**DOI:** 10.3201/eid1108.050110

**Published:** 2005-08

**Authors:** Astrid Vabret, Thomas Mourez, Julia Dina, Lia van der Hoek, Stéphanie Gouarin, Joëlle Petitjean, Jacques Brouard, François Freymuth

**Affiliations:** * University Hospital, Caen, France;; † University of Amsterdam, Amsterdam, the Netherlands

**Keywords:** phylogenetic analysis, coronavirus NL63, molecular detection, pneumonia, bronchiolitis, multiplex assay

## Abstract

The human coronavirus NL63 (HCoV-NL63) was first identified in the Netherlands, and its circulation in France has not been investigated. We studied HCoV-NL63 infection in hospitalized children diagnosed with respiratory tract infections. From November 2002 to April 2003, we evaluated 300 respiratory specimens for HCoV-NL63. Of the 300 samples, 28 (9.3%) were positive for HCoV-NL63. The highest prevalence was found in February (18%). The main symptoms were fever (61%), rhinitis (39%), bronchiolitis (39%), digestive problems (33%), otitis (28%), pharyngitis (22%), and conjunctivitis (17%). A fragment of the spike protein gene was sequenced to determine the variety of circulating HCoV-NL63. Phylogenetic analysis indicated that strains with different genetic markers cocirculate in France.

## Abstract 2

Coronavirus NL63 was found in hospitalized children with upper and lower respiratory infections.

Human coronaviruses (HCoVs) were first recorded in the late 1960s; they are associated mainly with respiratory tract illness but are also involved in enteric and central nervous system diseases. They are represented by 2 prototype strains, HCoV-229E and HCoV-OC43, which belong to antigenic groups 1 and 2, respectively. In 2003, human coronaviruses received worldwide attention with the emergence of severe acute respiratory syndrome (SARS) caused by a novel coronavirus (SARS-CoV). In 2004, the increase in research on theses viruses soon led to the discovery of 2 other human coronaviruses, HCoV-NL63 in the Netherlands and, more recently, CoV-HKU1 in China (1–3). In March 2004, van der Hoek et al. isolated HCoV-NL63 from a nasopharyngeal aspirate taken from a 7-month-old child hospitalized with bronchiolitis, conjunctivitis, and fever (1). One month later, Fouchier et al. characterized the same virus isolated from a nasal swab that had been collected from a child with pneumonia in April 1988 (2). Phylogenetic analysis showed that HCoV-NL63 is a new group 1 coronavirus, most closely related to HCoV-229E. Partial HCoV-NL63 sequences from Australia, Japan, and Canada have been submitted to the GenBank database, which indicates that this virus is distributed worldwide. Two retrospective studies were conducted in the Netherlands, and 11 additional HCoV-NL63–positive samples were detected from November 2000 to February 2003.

We tested for HCoV-NL63 in children with acute respiratory tract infection hospitalized in Caen from November 2002 to April 2003, described symptoms associated with this infection, and examined local strains for the genetic variability. We also evaluated a multiplex reverse transcription–polymerase chain reaction (RT-PCR) assay for classical coronaviruses as a tool to test human coronaviruses (except SARS-CoV).

## Materials and Methods

From November 2002 to April 2003, the virology laboratory (University Hospital, Caen, France) received 1,427 respiratory samples (nasal aspirates and swabs) from patients <20 years of age. All specimens were tested for influenza virus A and B; respiratory syncytial virus (RSV); parainfluenza virus 1, 2, 3, and 4; and adenovirus by direct or indirect immunofluorescence or virus isolation. Samples were also tested for human metapneumovirus (HMPV), rhinovirus, enterovirus, and HCoV 229E and OC43 by virus isolation and RT-PCR. A total of 556 samples (39%) were positive for any of these viruses. Symptoms indicated viral infection, as judged by the clinical department; therefore, samples were not tested for bacterial pathogens. Of the 556 positive samples, the following respiratory viruses were detected: RSV (37%, n = 205), rhinovirus (18%, n = 101), influenza virus A and B (15%, n = 86), HMPV (9.7%, n = 54), and HCoV-OC43 (1.2%, n = 7); no HCoV-229E were detected.

Of the 871 negative samples, 300 (50 per month) were tested for HCoV-NL63. These 300 samples represented 191 patients <2 years of age (64%), 46 patients 2–5 years of age (15%), and 63 patients 6–20 years of age (21%). All patients were hospitalized with acute respiratory tract illness. Data for 18 patients with recorded HCoV-NL63 infection were available and were examined retrospectively for specific respiratory symptoms. All patients consented to having their samples tested for respiratory viruses, including coronaviruses.

Two RT-PCR assays were used to detect HCoV-NL63 in respiratory samples. RNA was extracted by using the QIAamp Viral RNA Mini Kit (Qiagen, Hilden, Germany) according to manufacturer's instructions. The first RT-PCR assay was a 1-step simple RT-PCR that amplified a 255-bp fragment of the nucleocapsid (N) gene of HCoV-NL63 by using the following primers: N5-PCR2 (5´-GATAACCAGTCGAAGTCACCTAGTTC-3´) and N3-PCR2 (5´-ATTAGGAATCAATTCAGCAAGCTGTG-3´). The second assay was a 1-step multiplex RT-PCR that amplified the same 255-bp fragment of the N gene of HCoV-NL63, a 574-bp fragment of the membrane (M) gene of HCoV-229E, and a 334-bp fragment of the M gene of HCoV-OC43 by using previously described primers (4,5). These assays (OneStep RT-PCR kit, Qiagen) were undertaken in 25-μL reaction volume containing 2.5 μL RNA extract, 5 μL 5× Qiagen OneStep RT-PCR buffer, 1 μL 10 mmol/L deoxynucleoside triphosphate (dNTP), 1 μL Qiagen OneStep RT-PCR Enzyme Mix, 1.2 μL of 10 mmol/L each primer, 3 μL Qiagen OneStep RT-PCR kit Q solution, and RNase-free water to 25 μL. The reaction was carried out in a GeneAmp PCR system 2700 thermal cycler (Applied Biosystems, Foster City, CA, USA) with an initial reverse transcription step at 50°C for 30 min, followed by PCR activation at 95°C for 15 min, 40 cycles of amplification (30 s at 95°C, 30 s at 58°C, 1 min at 72°C), and a final extension step at 72°C for 10 min. Each RT-PCR test included water controls that were treated identically to the virus samples throughout and was performed with usual precautions to avoid contamination.

RT-PCR products were subject to electrophoresis on an agarose gel, stained with ethidium bromide, and visualized under UV light. The comparative analytical sensitivities of these simple and multiplex RT-PCR assays were previously studied on prototype strains by analyzing serial 10-fold dilutions of positive control for HCoVs NL63, OC43, and 229E. The analytical sensitivity was equivalent to detect HCoV-OC43, the multiplex assay was more sensitive (by one 10-fold dilution) to detect HCoV-229E, and less sensitive (by one 10-fold dilution) to detect HCoV-NL63 (data not shown). No cross-reaction of these tests was observed between these coronaviruses. Samples that were positive for HCoVs NL63, 229E, and OC43 were confirmed by using a DNA enzyme immunoassay (GEN-ETI-K DEIA, Sorin, Saluggia, Italy) carried out as recommended by the manufacturer with original probes previously described for HCoVs 229E and OC43 and the following probe defined in the N gene for HCoV-NL63: 5´-(Biotin)CCTCTTTCTCAACCCAGGGCTGATA-3´ (4). A third RT-PCR assay was carried out on 12 HCoV-NL63–positive samples amplifying a 523-bp fragment with spike (S) gene–specific primers NL63-S-sens (position 22557–22582: 5´-ACCGCTGTTAATGAGTCTAGATATG-3´) and NL63-S-antisens (position 23043–23063: 5´-GTCCTGCTATACGGCTTGAA-3´). This assay was performed essentially as described above. The RT-PCR products were purified by using ExoSAP-IT (USB Corporation, Cleveland, OH, USA) and sequenced with the primers by using the CEQ Dye Terminator Cycle Sequencing Quick Start Kit on a CEQ 8000 Genetic Analysis System (Beckman Coulter, Fullerton, CA, USA). The nucleotide sequences of the partial S gene (GenBank accession nos. AY994243–AY994254) were compared with the 2 prototype HCoV-NL63 sequences available in GenBank (NL63-Amsterdam1: NC005831 and NL: AY518894). Both nucleotide and predicted amino acid sequence alignments were prepared by using ClustalX version 1.83. The phylogenetic trees were constructed by using HCoV-229E as an outgroup.

## Results

**Figure 1:**
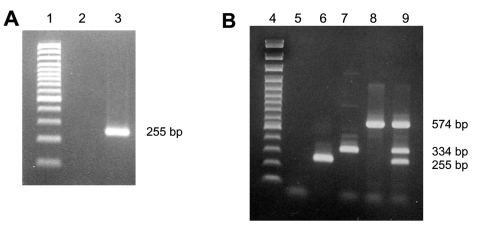
Ethidium bromide stain of 2% agarose gel showing reverse transcription–polymerase chain reaction (RT-PCR) products of human coronaviruses (HCoVs). A) Simple 1-step RT-PCR (HCoV-NL63, gene N): lane 1, size markers (100 bp); lane 2, negative control RT-PCR mix; lane 3, positive control HCoV-NL63. B) Multiplex 1-step RT-PCR (HCoVs NL63, OC43, 229E): lane 4, size markers (100 bp); lane 5, negative control RT-PCR mix; lane 6, positive control HCoV-NL63; lane 7, positive control HCoV-OC43; lane 8, positive control HCoV-229E; lane 9, mix of 3 positive control HCoVs (NL63, OC43, and 229E).

**Figure 2:**
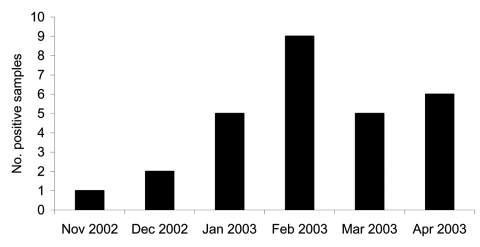
Number of human coronavirus NL63–positive samples per month. Fifty samples from patients hospitalized for acute respiratory symptoms were tested each month.

HCoV-NL63 was detected in 28 (9.3%) of the 300 samples evaluated from November 2002 to April 2003. Twenty-two samples were positive for HCoV-NL63 by both simple and multiplex RT-PCR (Figure 1). Discordant results were found for the remaining 6 samples: 3 were positive only in simple RT-PCR, and 3 were positive only in multiplex RT-PCR. These discordant samples were controlled by using the same methods from the RNA extraction product; the results obtained were identical. The specificity of the RT-PCR products under UV was confirmed by hybridization. Multiplex RT-PCR identified 3 samples with HCoV-OC43 and 1 sample with both HCoV-NL63 and HCoV-OC43. The 28 HCoV-NL63–positive samples were obtained from 18 patients <2 years of age (65%), 4 patients 2–5 years of age (14%), and 6 patients 6–15 years of age (21%). The age distribution of the patients infected by HCoV-NL63 was identical to the age distribution of the sample. Positive specimens were collected throughout the study period; no epidemic was observed. The temporal distribution of HCoV-NL63 infection is shown in Figure 2.

All patients included in this study had a respiratory tract illness. The medical reports of 18 patients with HCoV-NL63–positive samples were retrospectively examined, and the following symptoms were noted: fever (61%, n = 11), rhinitis (39%, n = 7), lower respiratory tract illness (bronchiolitis, pneumonia [39%, n = 7]), digestive problems (diarrhea and abdominal pain [33%, n = 6]), otitis (28%, n = 5), pharyngitis (22%, n = 4), and conjunctivitis (17%, n = 3). One patient had severe underlying disease (congenital immunodeficiency) and had upper respiratory tract illness with fever, another had a family history of atopic allergy, and pneumonia was diagnosed. Overall, more than one third of the patients infected by HCoV-NL63 had severe lower respiratory tract infection (6 bronchiolitis and 1 pneumonia). All of them recovered completely.

**Figure 3:**
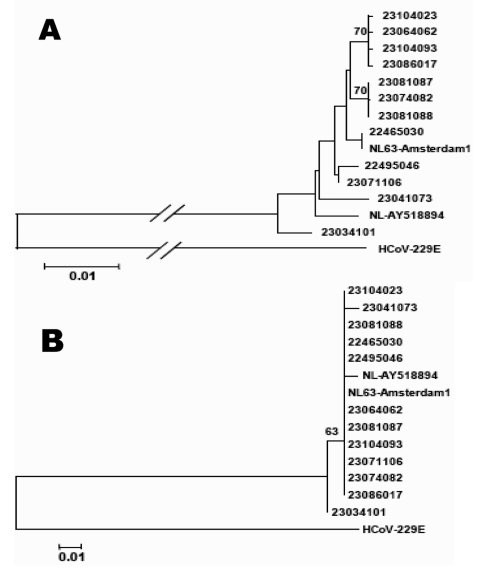
A) Phylogenetic analysis of a 523-bp region of the partial spike gene of 12 human coronavirus (HCoV)-NL63 isolates from France. To construct the trees, the 2 prototype strains NL63-Amsterdam1 and NL-AY518894 were included. HCoV-229E is used as an outgroup. A) Nucleotide sequence alignments created with ClustalX 1.83; bootstrap values ≥70 are indicated. B) Predicted amino acid sequences; bootstrap values ≥50 are indicated.

To determine if the isolates from France contain different genetic markers, we sequenced a part of the S protein gene of 12 isolates for molecular analysis. The phylogenetic analysis shows that the isolates from France are a divergent group containing sequences with different markers. One isolate (23034101) had characteristics of an outlier (Figure 3A). The phylogenetic analysis of the predicted amino acid sequence also shows that this isolate is an outlier (Figure 3B). However, the branches of this tree are based on only 1 amino acid difference. Care should be taken because of the limited informative sites in the sequence.

## Discussion

A number of viruses cause respiratory infections, and many infections cannot be attributed to any known pathogen (6). This fact may be because some detection methods lack sensitivity, because some respiratory viruses are not systematically tested for, or because some pathogens are not yet identified. Of the 4 novel agents, HMPV, SARS-CoV, HCoV-NL63, and CoV-HKU1, identified recently, 3 were coronaviruses (1,3,7–9). Coronaviruses infect many species of mammals and birds, they possess the largest genome of all RNA viruses (≈30 kb), and they have a high frequency of recombination. In addition, the potential to infect other species has been described for bovine coronavirus and is suspected to have caused the SARS outbreak (10,11). Therefore, coronaviruses represent a potential major infectious agent in humans. Based on genotypic and serologic characteristics, coronaviruses were divided into 3 distinct groups: with HCoVs 229E and NL63 in group 1 and HCoVs OC43 and HKU1 in group 2. SARS-CoV is not definitively assigned to any of these groups. However, an early split-off of SARS-CoV from the group 2 lineage was suggested (12). Until recently, only the HCoVs 229E and OC43 and SARS-CoV have been thoroughly studied. As suggested by epidemiologic surveys conducted in the 1970s, human coronaviruses are distributed worldwide and circulate during seasonal outbreaks (13–15). In this study, we determined whether this is also the case for HCoV-NL63. Furthermore, we looked at the symptoms of a HCoV-NL63 infection and the heterogeneity of the virus isolate circulating in France. In this study, 9.3% of the samples were positive for HCoV-NL63. These results suggest that HCoV-NL63 can frequently cause infections, particularly in young children.

Because of availability, only samples that tested negative for other respiratory viruses were included in this study. Consequently, we could not identify co-infections. Nevertheless, by using multiplex RT-PCR, which simultaneously detected the classical human coronaviruses (OC43, 229E, and NL63), we detected 1 co-infection by HCoVs NL63 and OC43 and 3 HCoV-OC43–positive samples. These additional cases of HCoV-OC43 infection in our patients can be explained by the fact that multiplex RT-PCR was performed directly on respiratory samples, whereas in previous routine tests, samples were first instilled into a cell culture system (HUH7 cell line) and RT-PCR was then performed on cell culture supernatant.

Detecting human respiratory coronaviruses requires molecular techniques because of difficulties in virus culture and lack of an assay to detect intracellular antigens or others serologic assay. A multiplex RT-PCR is therefore a useful tool to simultaneously test for various HCoVs from a clinical sample. This study showed that the clinical sensitivity of multiplex RT-PCR was equivalent to simple RT-PCR, allowing clinical studies and routine testing.

No HCoV-229E was detected in samples. HCoVs 229E and NL63 both belong to antigenic group 1. The percentage amino acid sequence identities between the S, M, and N proteins of HCoVs NL63 and 229E are 54.7%, 61.5%, and 43.2%, respectively (2). A cross-protective immune response could explain why these 2 human coronaviruses do not circulate at the same time. We detected HCoV-NL63 in respiratory specimens in February with a frequency of 18%. These results correlate with the fact that human coronaviruses circulate primarily in the winter. However, HCoV-NL63 was found in nasal aspirates each month of our study.

The clinical symptoms associated with HCoV-NL63 still need to be determined. In the patients in our study, symptoms included not only respiratory symptoms but also lower respiratory tract diseases such as bronchiolitis, bronchitis, and pneumonia. Human coronaviruses, except SARS-CoV, generally cause disease much like the common cold, but they have also been associated with more severe lower respiratory tract conditions, especially in frail patients (4,16). Whether HCoV-NL63 is also responsible for coldlike illnesses in healthy adults, as has been described for HCoVs 229E and OC43, must be determined. The same can be said about the very recently described coronavirus CoV-HKU1. This virus was identified in a 71-year-old patient with chronic obstructive airway disease who was hospitalized with pneumonia (3).

Digestive problems were noted in approximately one third of patients. No clear evidence exists that human coronaviruses, except SARS-CoV, cause enteric illness, but previous studies have suggested that these viruses may be involved in enteric diseases (17–20). Further studies must be conducted to detect coronaviruses in stool samples and clarify the origin of these digestive symptoms.

The S protein of coronaviruses is a major determinant of cell tropism and pathogenicity and a major inducer of neutralizing antibodies (21). Furthermore, heterogeneity of the S gene has been observed for different HCoV-NL63 strains. We amplified part of the S gene to study the variability of our isolates. Phylogenetic analysis showed that several different isolates are cocirculating in France, similar to the situation in the Netherlands, Australia, Canada, and Belgium.

In conclusion, HCoV-NL63 can be found in patients with upper and lower respiratory tract illness, particularly in hospitalized children. This observation is the first of HCoV-NL63 infection in France, and several isolates of HCoV-NL63 were found to circulate in our country. The sensitive multiplex RT-PCR for the HCoVs NL63, 229E, and OC43 that we developed is a useful tool to facilitate the routine detection of these pathogens.

This project was supported by the European Commission EPISARS contract (No. SP22-CT-2004-511063) and the Programme de Recherche en Réseaux Franco-Chinois (épidémie du SRAS: de l'émergence au contrôle).
